# Addison's Disease and Dilated Cardiomyopathy: A Case Report and Review of the Literature

**DOI:** 10.1155/2016/4362514

**Published:** 2016-11-28

**Authors:** Viktoriya Mozolevska, Anna Schwartz, David Cheung, Bilal Shaikh, Kapil M. Bhagirath, Davinder S. Jassal

**Affiliations:** ^1^Institute of Cardiovascular Sciences, St. Boniface General Hospital, University of Manitoba, Winnipeg, MB, Canada; ^2^Section of Cardiology, Surrey Memorial Hospital, University of British Columbia, Vancouver, BC, Canada; ^3^Section of Cardiology, Department of Internal Medicine, University of Manitoba, Winnipeg, MB, Canada; ^4^Department of Radiology, St. Boniface General Hospital, University of Manitoba, Winnipeg, MB, Canada

## Abstract

Addison's disease is often accompanied by a number of cardiovascular manifestations. We report the case of a 30-year-old man who presented with a new onset dilated cardiomyopathy due to Addison's disease. The clinical presentation, treatment, and outcomes of this rare hormone mediated cardiac disorder are reviewed.

## 1. Introduction

Addison's disease, also known as primary adrenal insufficiency, is associated with a decreased production of glucocorticoid and mineralocorticoid hormones from the adrenal cortex [1]. Although autoimmune adrenalitis is considered to be the major cause of Addison's disease in up to 90% of diagnosed individuals, prevalent in female patients between 30 and 50 years of age, other etiologies include infectious, drug induced, and/or genetic factors [2, 3]. Common manifestations of this condition are hyponatremia, hyperkalemia, and/or hypoglycemia along with mucosal and skin hyperpigmentations [2, 3]. Although cardiovascular manifestations of Addison's disease include hypotension, syncope, and arrhythmias, the development of a dilated cardiomyopathy and heart failure are an uncommon life-threatening complication [4–8]. We describe a rare case of a 30-year-old male with a dilated cardiomyopathy in the context of Addison's disease and review all 6 adult cases of this cardiac manifestation published to date [4–8]. We evaluate the clinical presentation, treatment, and outcomes of these cases and discuss the diagnosis, pathophysiology, and treatment of this rare hormone mediated cardiac disorder.

## 2. Case Report

A 30-year-old previously healthy male presented to the emergency department with constitutional symptoms of weakness, fatigue, dizziness, and anorexia. A baseline EKG and chest X-ray were within normal limits. He was diagnosed with primary adrenal insufficiency presenting with biochemical evidence of high ACTH (>278 pmol/L) and low cortisol levels (32 nmol/L) and started on prednisone 5 mg po od and fludrocortisone 0.1 mg po od. Two weeks later, he presented with a 5-day history of orthopnea and increasing dyspnea on minimal exertion. On presentation, the blood pressure was 104/74 mm Hg with a heart rate of 90 beats per minute. The jugular venous pressure was elevated at the angle of the jaw with displacement of the cardiac apex and normal heart sounds. There were diminished breath sounds bilaterally in both lower lung fields without evidence of peripheral edema. The serum sodium was mildly reduced at 132 mmol/L (normal range: 135–145 mmol/L) while the potassium was increased at 5.6 mmol/L (normal range: 3.5–5.0 mmol/L). There was a markedly elevated N-terminal brain natriuretic peptide of 4515 pg/mL (normal range <125 pg/mL), consistent with clinical and biochemical evidence of acute heart failure. The serum TSH, urine toxicology, and metanephrines were within normal limits. A 12-lead EKG demonstrated poor R-wave progression across the precordial leads with evidence of a left anterior fascicular block. Chest X-ray findings were consistent with bilateral pleural effusions, vascular redistribution, and interstitial edema. Transthoracic echocardiography (TTE) confirmed a dilated left ventricle (LV), severe LV systolic dysfunction with an ejection fraction of 15%, and moderate functional mitral regurgitation. Cardiac magnetic resonance imaging with late gadolinium enhancement was normal with no evidence of a viral myocarditis nor infiltrative cardiomyopathy. Cardiac catheterization demonstrated normal coronaries consistent with the diagnosis of a nonischemic dilated cardiomyopathy. A computed tomographic scan of the abdomen revealed hyperdense adrenal glands with central necrotic areas of hypoattenuation and peripheral enhancement, consistent with the recent diagnosis of primary adrenal insufficiency. With a new diagnosis of acute heart failure due to primary Addison's disease, the patient was admitted for medical treatment with parenteral diuretics, ACE inhibition, beta blockade, and a decrease in the fludrocortisone dosage (that was started 2 weeks before). A 6-month follow-up multigated acquisition scan (MUGA) revealed an improvement in LV ejection fraction to 40–45%.

## 3. Discussion

With an incidence of approximately 120 cases per million in the population, Addison's disease is a rare long term endocrine disorder in which the adrenal glands produce insufficient steroid hormones, including glucocorticoids and mineralocorticoids [1–3, 9]. The clinical presentation of Addison's disease encompasses a multitude of systemic symptoms including fatigue, weight loss, salt craving, and joint and back discomfort [1–3, 9]. Individuals with primary adrenal insufficiency may also experience a number of cardiovascular symptoms including hypotension, arrhythmias, and congestive heart failure [4–8]. In fact, the development of a dilated cardiomyopathy due to Addison's disease is extremely rare [4–8]. Although the exact etiology of heart failure due to Addison's disease remains unknown, a number of potential mechanisms include (i) reduced effects of cortical hormones on the LV myocardium; (ii) poor alimentation with low glycogen reserves; (iii) hemoconcentration, low blood volume, and reduced coronary blood flow; and (iv) disturbances in electrolyte levels [9].

After a systematic review of the literature of all adult cases of Addison's disease and dilated cardiomyopathy, a total of 6 published cases (including the current report) were identified as shown in Table 1 [4–8]. The mean age was 38 years (range 21–53) with a male to female ratio of 2 : 1. Common cardiovascular features at presentation were hypotension, dyspnea, tachycardia, and pulmonary edema. Our case was unique in that the patient developed bilateral pleural effusions and the lowest LVEF as measured by TTE. Moreover, our case was only the second in which a patient developed symptoms of acute CHF following administration of hormone replacement therapy (HRT) and the one with the shortest time lapse between the two events of only 6 days (Table 1) [4–8].

In patients with Addison's disease, dual HRT with glucocorticoids and mineralocorticoids is a necessity [1–3, 10]. Although oral hydrocortisone and fludrocortisone are the mainstay of treatment in this patient population [1–3, 10], the use of these medications may need to be altered in the setting of a concomitant heart failure crisis. In particular, fludrocortisone corrects hypotension by increasing sodium retention, thereby increasing systemic afterload. However, in the presence of a dilated cardiomyopathy and reduced LVEF, increased afterload by fludrocortisone may precipitate acute congestive heart failure [4–8]. In individuals with Addison's disease who experience acute cardiovascular symptoms due to the development of a dilated cardiomyopathy, the dosage of fludrocortisone may need to be decreased or stopped altogether in order to correct the acute heart failure syndrome. In 4 of the 6 cases reviewed in Table 1, the patients were able to tolerate a carefully balanced dose of fludrocortisone after appropriate treatment of the underlying heart failure symptoms with medical therapy including diuretics, ACE inhibition, and beta blockade [4–8]. These agents, while appropriate for acute heart failure in the setting of a reduced LVEF, may interfere with the effectiveness of fludrocortisone in correcting hormonal deficiency, necessitating careful hemodynamic monitoring of the patient's condition throughout the treatment period.

## 4. Conclusion

A multidisciplinary approach must be sought in the management of patients with dilated cardiomyopathy secondary to Addison's disease. HRT dosage should be optimized, heart failure therapy should be initiated, and patients should be closely monitored for future cardiovascular complications.

## Figures and Tables

**Table 1:** Summary of adult case reports of Addison's disease and dilated cardiomyopathy.

Case (reference)	Report year	Age/sex	CV manifestations	LVEF (%)	Treatment	Outcome
Cushner et al. [4]	1963	53/M	Dyspnea, orthopnea, LV enlargement, pulmonary edema, and right pleural effusion	N/A	(1) Meralluride, anticoagulation therapy, quinidine, digitalis (2) ACTH, 20 units (3) 9-alpha-Fluorohydrocortisone, 0.2 mg 3 times daily for 7 days prior to death	Day 20—decrease in pulmonary congestion Day 31—patient died
Bhattacharyya and Tymms [5]	1998	47/F	Edema, dyspnea, tachypnea, lung crepitations, cardiomegaly, and pulmonary congestion	N/A	(1) IV furosemide for 6 days (2) Fludrocortisone dosage decreased by 50% (from 30 mg daily) and then stopped after 2 days	(i) CHF symptoms resolved (ii) Addisonian crisis at 8 months, treated with hydrocortisone and fludrocortisone (iii) At 12 months, patient was doing well
Afzal and Khaja [6]	2000	36/M	Dyspnea, fatigue, dizziness, malaise, and tachycardia	25%	(1) Corticosteroid therapy (2) No treatment for CHF	(i) At 7 weeks, LVEF improved to 55%
Wolff et al. [7]	2007	42/F	Hypotension, tachycardia, respiratory failure, bilateral pulmonary edema, and cardiogenic shock	30%	(1) IV hydrocortisone loading dose 100 mg and thereafter 10 mg/h (2) Mechanical ventilation and continuous norepinephrine at 4.4 *μ*g/kg min (3) At discharge, oral hydrocortisone and fludrocortisone, standard	(i) At discharge, the LVEF improved to 52% with no evidence of wall-motion abnormalities
Krishnamoorthy et al. [8]	2013	21/M	Nausea, weakness, progressive dyspnea, asystolic cardiac arrest, pericardial effusion, severe biventricular failure, and cardiogenic shock	N/A	(1) TandemHeart implantation (2) IV hydrocortisone loading dose 100 mg and thereafter 50 mg/8 h (3) Weaned to physiologic dose of oral hydrocortisone by discharge (4) 2 months after discharge, mineralocorticoid replacement for hypotension	(i) At 2 weeks after RVAD and LVAD removal, normal biventricular function (ii) At 2 months, remained well
Mozolevska et al.	2016	30/M	Orthopnea, dyspnea, elevated jugular venous pressure, elevated BNP, dilated LV, severe systolic dysfunction, and bilateral pleural effusions	15%	(1) IV furosemide, 40 mg oral at discharge (2) Ramipril 5 mg (3) Bisoprolol 10 mg (4) Fludrocortisone dosage decreased by 50% (from 0.1 mg daily), prednisone unchanged at 5 mg daily	(i) LVEF improved to 44%

LV, left ventricle; IV, intravenous; CHF, congestive heart failure; LVEF, left ventricular ejection fraction; ICU, intensive care unit; RVAD, right ventricular assist device; LVAD, left ventricular assist device; BNP, brain natriuretic peptide.
